# Adding Value to Bycatch Fish Species Captured in the Portuguese Coast—Development of New Food Products

**DOI:** 10.3390/foods10010068

**Published:** 2020-12-31

**Authors:** Frederica Silva, Ana M. Duarte, Susana Mendes, Patrícia Borges, Elisabete Magalhães, Filipa R. Pinto, Sónia Barroso, Ana Neves, Vera Sequeira, Ana Rita Vieira, Maria Filomena Magalhães, Rui Rebelo, Carlos Assis, Leonel Serrano Gordo, Maria Manuel Gil

**Affiliations:** 1 MARE—Marine and Environmental Sciences Centre, Polytechnic of Leiria, Cetemares, 2520-620 Peniche, Portugal; frederica.g.silva@ipleiria.pt (F.S.); ana.c.duarte@ipleiria.pt (A.M.D.); filipa.gomes@ipleiria.pt (F.R.P.); sonia.barroso@ipleiria.pt (S.B.); 2 MARE—Marine and Environmental Sciences Centre, Faculdade de Ciências, Universidade de Lisboa, 1749-016 Lisbon, Portugal; eli_magalhaes_silva@hotmail.com (E.M.); amneves@fc.ul.pt (A.N.); vlsequeira@fc.ul.pt (V.S.); arivieira@fc.ul.pt (A.R.V.); caassis@fc.ul.pt (C.A.); lsgordo@fc.ul.pt (L.S.G.); 3 MARE—Marine and Environmental Sciences Centre, ESTM, Polytechnic of Leiria, Cetemares, 2520-620 Peniche, Portugal; susana.mendes@ipleiria.pt (S.M.); patricia.borges@ipleiria.pt (P.B.); 4 Departamento de Biologia Animal, Faculdade de Ciências, Universidade de Lisboa, 1749-016 Lisbon, Portugal; mfmagalhaes@fc.ul.pt (M.F.M.); rmrebelo@fc.ul.pt (R.R.); 5 CE3C—Centre for Ecology, Evolution and Environmental Changes, Faculdade de Ciências, Universidade de Lisboa, 1749-016 Lisbon, Portugal

**Keywords:** fish valorization, consumer, unexploited, low commercial value, hedonic tests, sustainability, product reformulation

## Abstract

We live in a world of limited biological resources and ecosystems, which are essential to feed people. Consequently, diversifying target species and considering full exploitation are essential for fishery sustainability. The present study focuses on the valorization of three low commercial value fish species (blue jack mackerel, *Trachurus picturatus*; black seabream, *Spondyliosoma cantharus*; and piper gurnard, *Trigla lyra*) and of two unexploited species (comber, *Serranus cabrilla* and boarfish, *Capros aper*) through the development of marine-based food products with added value. A preliminary inquiry with 155 consumers from Região de Lisboa e Vale do Tejo (Center of Portugal) was conducted to assess fish consumption, the applicability of fish product innovation, and the importance of valorizing discarded fish. Five products (black seabream *ceviche*, smoked blue jack mackerel pâté, dehydrated piper gurnard, fried boarfish, and comber pastries) were developed and investigated for their sensory characteristics and consumer liking by hedonic tests to 90 consumers. The most important descriptors were identified for each product (texture, flavor, color, and appearance). Comber pastries had the highest purchase intention (88%), followed by black seabream *ceviche* (85%) and blue jack mackerel pâté (76%). Sensory evaluations showed a clear tendency of consumers to accept reformulated products, with the introduction of the low-value and unexploited species under study.

## 1. Introduction

The growing world population and, consequently, the increasing need for food resources are demanding a more sustainable and circular bio-economy. In fact, the planet’s sustainability is one of the most discussed topics worldwide, with particular emphasis on marine resources. Despite the ocean’s richness in different fish species, consumers and industries only know a few of them, which are the ones with the highest commercial value. However, underexploited and underutilized fish species may offer added benefits for consumer health, given that their nutritional content as well as sensory attributes are equally pleasant as more commonly consumed fish [1]. 

Fish species that are not targeted by fisheries are called bycatches, which means (a) species with low or no commercial value that are discarded to gain room on the vessel for commercial species caught later; (b) discarded species with no commercial value due to restrictive measures in terms of landing (quota, size below the minimum conservation reference size); (c) deteriorated species; or (d) protected species that cannot be caught, which in most cases do not survive [2,3]. Fish disposal results in marine ecosystem changes, which can be avoided with the exploitation of waste from bio-based industrial processes, including by-products, co-products, and discards (from fisheries), contributing to resource-efficient process improvement [4]. 

In Portugal, the most consumed fish species are cod (*Gadus morhua*), hake (*Merluccius merluccius*) [1], and canned tuna (made with some species from the Scombridae family) [5,6]. On the other hand, in Europe the top five consumed species, which accounted for 44% of the total volume in 2017, were tuna, cod, salmon, Alaska pollock, and shrimp [7]. This consumption preference can be explained by the lack of consumer awareness of other species, which discourages their purchase. However, this may not be the only explanation, due to the complexity of food choice, which is influenced by many interrelated factors, including social, environmental, political, economic, and cultural aspects [8]. According to Coralo et al. [8] consumers’ attitude towards food can be categorized as (a) the Individualist: food choices based on their personal interests (e.g., economic convenience, personal mood); (b) the Foodie: food choices based on the sensory aspects related to food (e.g., better taste, right price–quality ratio); (c) the Environmentalist: food choices based on environmental sustainability issues (e.g., the integrity of the farmer, food origin); and (d) the Health Enthusiast: food choices based on contemporary diets (e.g., low-calorie diet) and analyzing label contents, claims, and health effects. Therefore, with consumer surveys, it is possible to quantify and measure consumption options and frequencies, which allows a pattern of eating behavior to be traced. Regardless of the population’s consumption habits, one of the ways to make a new fish species known is through its use in traditional products or in products that are familiar to the consumer, replacing the more commercial species. After acknowledging population consumption habits and expectations regarding new food products, this information is applied to product development and, subsequently, sensory tests are carried out to assess acceptability and purchase intention. Sensory evaluation allows for the establishment of a target market, the identification of the most important product features the avoidance of wasted effort during product development, quality issues to be dealt with; a comparison between brands, and an attempt at ensuring long shelf-life [9]. Therefore, sensory evaluation is a result of human decision and, consequently, it is an outcome of complex interactions conditioned by personal history, environmental variables, subjective covariates, and object characteristics that also interact with the modality of the survey [9]. In fact, sensory evaluation is essential for both new food product development and food reformulation of existing products. 

Food reformulation is considered one of the contributors to achieving population nutrient goals (e.g., energy and salt reduction, clean labels) [10,11]. Therefore, food reformulation can be a way to valorize fish species with low or no commercial value by using them to replace the commercial ones. This both increases the value of underexploited and underutilized fish species and reduces the final price of the reformulated product, with the added advantage of avoiding overexploitation of species with significant commercial value. 

When considering development of new fish-based food products, attention should be given to seasonality to achieve greater nutritional (e.g., fat and protein content) and sensorial (e.g., appearance, odor, flavor, and texture) features. Therefore, it is important to identify the best time of the year to capture fish considering the product formulation and, consequently, consumer acceptance, taking into consideration their life history, including their reproductive cycle and growth characteristics. Considering that there is a high probability of market failure when introducing new food products, it is crucial to know in advance the consumer habits and preferences to create or redesign and reformulate a product that satisfies their necessities [12]. One of the approaches to gain this knowledge is to perform a market survey about consumers’ diet and the reason for their food choices (e.g., nutritionally rich, easy to prepare). Consumer characterization (e.g., gender, age, occupation) is also important to identify a potential target market segment. 

Despite world fisheries and aquaculture production increasing in the last 30 years from 100 million tonnes in 1986 to 178 million tonnes in 2018 [13], fisheries showed a stable value around 90 million tonnes between 1996 and 2016 and a slight increase to 96 million tonnes in 2018 [13]. However, regardless of this stability in catches, the number of unsustainable (overfished) stocks has increased from 10% in 1974 to 34.2% in 2017. In addition, within the sustainable stocks, the number of underfished stocks decreased continuously from 1974 (around 38%) to 2017 (less than 8%), with the majority of stocks in the “maximally sustainably fished” category [13]. In Portugal, as in other countries from temperate waters, most fisheries are multi-specific, meaning that besides the target species (usually species with high commercial value) other species are also caught. Among these, some are discarded at sea and others are landed but normally reach a low commercial value. In terms of stock status, many commercial species of more global (European) interest are studied and their stocks are assessed and managed by international organizations (International Council for the Exploration of the Sea—ICES). The other species, the great majority of them, namely those included in this paper, are not assessed and managed and the biological information is scarce and very often totally lacking. The minimization of discards and the valorization of non-commercial or low commercial species is therefore very important to present alternatives to the currently most exploited species. The inclusion of new alternative species in the market would allow the increase of first auction prices and then the interest of fishermen to shift their effort to these species, as occurred in the 1990s with the black scabbardfish fishery in Portuguese waters.

Therefore, the present study aims to value bycatch species with low commercial and non-commercial value. Among the former, three low commercial fish species were chosen: blue jack mackerel (*Trachurus picturatus*), black seabream (*Spondyliosoma cantharus*) and piper gurnard (*Trigla lyra*). Why these species? These species are particularly relevant either because of their high landing values or their first auction prices. Among species of non-commercial value, the comber (*Serranus cabrilla*) and the boarfish (*Capros aper*) are particularly abundant in Portuguese waters, with *S. cabrilla* being one of the most important species in terms of discards of several fisheries, namely gillnets and trawls, while *C. aper* is among the 10 most important species in terms of abundance, being caught as bycatch in both crustacean and fish trawl fishing [14,15]. The valorization of these species is aimed at through the development of five innovative and differentiating marine-based products in relation to what currently exists on the market. A market survey about fish consumption habits, the applicability of fish product innovation, and the importance of valuing discarded fish was also performed.

## 2. Materials and Methods

### 2.1. Market Survey—Fish Consumption, Applicability of Fish Products Innovation, and Importance of Fish Valorisation with Low or Absent Commercial Value 

A total of 155 consumers from Região de Lisboa e Vale do Tejo (Center of Portugal), answered to an anonymous market survey with 13 closed-ended questions divided into three groups: (1) consumer baseline characteristics, (2) fish consumption habits and their characterization, and (3) individual consumer preference and consumption patterns of processed fish products. In the first group, the consumer’s personal features (gender, age, level of education, and occupation) were identified. The second group was conceived to assess individual importance of fish consumption (rated on a scale of 5—Very important, 4—“Important”, 3—“Indifferent”, 2—“Less important,” and 1—“Not important”), the main attributes that are valorized in fish consumption (e.g., low fat content, protein, flavor), consumption frequency characterization (rated on a scale of 5—“Occasionally,” 4—“More than once a week,” 3—“Every week,” 2—“Twice a month,” and 1—“< once a month”), and to perceive the consumption of some fish species with low or no commercial value. The third group allowed for the appraisal of the willingness to consume new products derived from fish species with low or no commercial value, the main motivations for its consumption (e.g., low-price products, fast and practical products, nutritionally rich), how to consume them (e.g., to prepare in the oven, ready-to-eat products, or frozen products), an evaluation of the importance given to their valorization (rated on a scale of 5—“Very important,” 4—“Important,” 3—“Indifferent,” 2—“Less important,” and 1—“Not important”), and the importance of creating new fish products from them (rated on a scale of 5—“Very important,” 4—“Important,” 3—“Indifferent,” 2—“Less important,” and 1—“Not important”). This data collection, through a market survey, was done personally and ran from September to November 2019. 

### 2.2. Fish-Based Food Product Production and Preparation 

The fish species used in this work were blue jack mackerel, black seabream, piper gurnard, comber, and boarfish. The fish (captured on the Portuguese coast) were acquired at Peniche fish auction or purchased from local fishermen. The initial fish preparation included the removal of scales, internal organs, and head. 

The fish products were developed by a Portuguese chef, considering the consumers’ answers to the survey, and the need to be sensorially pleasing and preferably ready-to-eat or with few preparation requirements. In addition, no food additives were added to any fish product. Given the consumers’ lack of knowledge about the fish species under study, it was decided to develop reformulations of products familiar to consumers. In addition, new consumption trends were also considered. 

The black seabream used in ceviche formulation was initially filleted, the skin was removed, and then sliced into small cubes. The black seabream cubes were then mixed with the remaining ingredients, which included lime, lemon, chives, vinegar, ginger, mango, and red pepper. 

For the preparation of the smoked blue jack mackerel pâté, the fish fillets were smoked with bay leaves, the skin was removed, they were minced, and then added to the remaining ingredients that included chili, cream cheese, pickles, onion, and mustard.

Piper gurnard skinless fillets were first deep-fried with vegetable oil and, after excess oil removal, the fish was dehydrated.

For the product formulation, the boarfish was first scaled, leaving the caudal fin, and then coated with flour and fried in vegetable oil. 

For the preparation of the comber pastries, the fish was smoked with bay leaves, minced, and added to the other ingredients: mashed potato, onion, parsley, and egg. After mixing all the ingredients the pastries were molded with the help of spoons and deep-fried.

### 2.3. Hedonic Tests

Each of the fish products developed was evaluated by 90 consumers of both genders aged between 18 and 75 years old with a hedonic sensorial test at the “European night of researchers” event in Lisbon, Portugal. The aim was to measure the overall hedonic perception of a product by consumers. This test had 9 closed-response questions and was divided into three groups: (1) consumer characterization (gender and age), (2) product sensorial evaluation (general appearance, color, odor, flavor, texture, and global appreciation), and (3) purchase intention. The sensorial evaluation was performed considering a scale of 9 (“Extremely pleasant”), 8 (“Very pleasant”), 7 (“Pleasant”), 6 (“Not very pleasant”), 5 (“Neither pleasant nor unpleasant”), 4 (“Not very unpleasant”), 3 (“Unpleasant”), 2 (“Very unpleasant”), and 1 (“Extremely unpleasant”), and the purchase intention from 5 (“I’m sure I would buy”), 4 (“I would probably buy”), 3 (“I don’t know if I would buy”), 2 (“I probably wouldn’t buy”) and 1 (“I’m sure I wouldn’t buy”).

### 2.4. Statistical Analysis 

Descriptive statistics and exploratory data analysis were performed on 155 consumers sampled in the same region to characterize the fish consumer profile, the applicability of fish product innovation, and the importance of valorizing discarded fish. An exploratory factorial analysis (EFA) was performed to achieve a parsimonious representation of the associations among measured descriptors (that is, appearance, color, odor, flavor, texture, and global appreciation). Prior to running the EFA, the uniformity of the sample was tested by the Kaiser–Meyer–Olkin (KMO) measure of sampling adequacy. A KMO value of more than 0.50 was considered acceptable. In addition, the measure of sampling adequacy of the anti-image correlation matrix was all greater than 0.5. The presence of correlations between measured descriptors was tested using the Bartlett test of sphericity and was accepted when it was significant at *p*-value < 0.05. The correlation matrix was also analyzed and values of more than 0.3 were considered suitable [16]. As regards the sample size, there are several guiding rules cited in the literature [16,17,18] and the lack of agreement is noted in all of them. In this work, it was an option to follow the recommendation denoted as a ratio of N:*p*, where N refers to the number of participants and *p* to the number of variables. Thus, the ratio of respondents to variables should be at least 10:1 [19], which was satisfied by the data set under study. Principal component analysis (PCA) was used for factor extraction and orthogonal rotation (varimax option) to minimize the number of indicators that have high loading on one factor [20]. The number of factors was retained based on the eigenvalue >1.0 criterion (Kaiser criterion) and a plot of the eigenvalues, which shows the total variance associated with each other [21]. For all analyses, this resulted in the extraction of the first two factors.

Spearman’s rank–order correlation coefficient was used to assess the co-variation of the descriptor scores. The Kruskal–Wallis non-parametric test was performed to assess the statistical differences between descriptor scores and purchase intention when comparing age groups. When significant differences were achieved, the Games–Howell or Bonferroni multiple comparison tests were executed. The use of the Kruskal–Wallis test proved to be appropriate, since it allows for the comparison of distributions of two or more ordinal variables less observed in two or more independent samples. Finally, the Mann–Whitney non-parametric test was performed to assess the statistical differences between descriptors and purchase intention when comparing consumer gender. 

All analyses were performed for the five fish-based products that were developed. The significance level was set to *p*-value < 0.05. Analyses were carried out using IBM SPSS Statistics 26 software.

## 3. Results and Discussion

### 3.1. Market Survey

The respondents from the market survey were mostly employed (57%), doctoral (33%), women (69%), and aged between 31 and 45 (42%) (Table 1). The participants give high importance to fish consumption (72%), mainly due to their fatty acid and omega content (79%) (Table 2). Portugal is among the countries with the highest consumption of fish (about 60 kg per capita per year [13]) and the sample reflected this tendency with fish consumption every week (44%) or more than once a week (43%), i.e., 87% of respondents often consumed fish, some of which with low or absent commercial value (48%) (Table 2). The consumption of processed fish products was present in the consumers’ diet (75%) due to their quick and easy preparation (38%). In addition, the consumers were receptive to new products (86%), preferably frozen, ready-to-eat, or to be prepared in the oven (51%) (Table 3). These findings on nutritional fish factors and purchasing convenience were also reported by Samoggia and Castellini’s study developed in Italy [22]. In their study, 740 consumers were asked about fish frequency consumption, with a focus on healthy attitude and sociodemographic or family structure characteristics [22]. Their results showed that the consumption frequency was mostly driven by health-orientation and social influences, compared with socio-economic characteristics (including households with children) [22].

The consumers give a major importance to fish valorization (61%), especially through new food product formulation (54%) with proper promotion (57%) (Table 3). Although the number of respondents was not representative of the Portuguese population, this study allowed for a preliminary perception of their receptivity to new fish products and the expectations consumers have of these products.

The importance of ecological sustainability with respect to seafood was also reported [23], where more than half of the interviewees demonstrated good understanding about this topic and were willing to pay more to improve them. 

In a systematic review the relation between fish consumption and nutritional benefits, especially omega-3 fatty acids, was also reported [24]. In addition, the authors considered a number of barriers to fish consumption, where the most important was related to sensory disliking of fish, lack of convenience, lack of self-efficacy in selecting and preparing fish, health risk concerns, lack of fish availability, and high prices [24]. These authors also reported that country and origin, production and preserving methods, product innovation, packaging, and eco-labelling were the most relevant fish attributes that affected consumers’ choice [24]. Therefore, rapid and effective responses are needed to provide new insights for major actors (fishermen and policymakers) to improve the environmental sustainability of fish consumption [24].

### 3.2. Fish-Based Developed Food Products 

**Figure 1:**
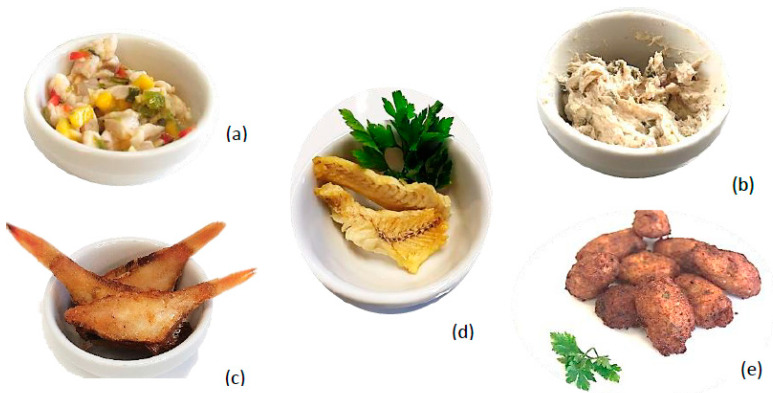
Reformulated fish products developed with each species. Legend: (**a**) black seabream ceviche, (**b**) smoked blue jack mackerel pâté, (**c**) fried boarfish, (**d**) dehydrated piper gurnard, and (**e**) comber pastries. Adapted from [1].

The fish products developed during this study were black seabream ceviche, smoked blue jack mackerel pâté, dehydrated piper gurnard (chip-like treats), fried boarfish, and comber pastries (Figure 1). Black seabream ceviche, smoked blue jack mackerel pâté, and dehydrated piper gurnard are ready-to-eat products, whereas breaded boarfish and comber pastries are both products to be sold frozen and ready-to-fry. 

When developing new fish-based products, the season of capture may be an important factor as the fish may have different nutritional composition, many times related with the reproductive cycle, which influences the fat reserves of the fish and thus creates different sensory perceptions when consumed. Hence, for the product formulations, the previous work of Silva et al. [25], related to the sensorial characterization of fresh fish species throughout the year, was taken into consideration [25]. 

Ceviche development should be carried out with a fish species with cohesion, chewability, and stiffness, which give a favorable texture to the product. In addition, an ivory color is also an advantage to the product appearance, enabling the fish to be highlighted. Thus, considering our previous work [25], the black seabream should be caught during winter, when it is characterized by sea, butter, and seaweed odors, which are also good features for this product. 

The blue jack mackerel, used in pâté formulation, should be caught during winter, especially due to the texture, flavor, and odor features related to that season, namely chewability, cohesion, and stiffness, as well as seaweed odor, sea odor, and flavor, which enhance the pâté sensory experience [25]. 

Piper gurnard should be caught during autumn due do its fat content, despite the absence of descriptors with statistical significance in our previous work [25]. In addition, piper gurnard captured in this season is characterized by a white color, contributing to a better fry product appearance [25].

The boarfish should be caught during winter due do its texture attributes, namely its cohesion, stiffness, and chewability properties, which allow texture maintenance after frying [25]. The comber fish was captured during winter due to its “ideal” stiffness, also being related to the laminar structures and sweet taste at the end of this season, which are essential to enhancing the fish flavor in the final product [25]. 

Regardless of the sensorial features, other factors should be considered to identify the most favorable season to catch the fish species under study, such as sexual cycle and abundance. 

Fish is a food source with different beneficial nutrients for human health, including fatty acids, proteins, vitamins, and mineral elements [26]. However, food processing methods are related to nutrient loss in an extent that depends on, for example, temperature and pressure due to chemical and physical changes [27]. Therefore, when different ingredients are combined, there is a formulation enrichment that is a way of valorizing certain foods such as fish. 

The valorization of a food ingredient through the development of new products or by the reformulation of existing ones allows the consumer to be more receptive to buying them, especially when the ingredient to be valorized is a discarded fish species with an uncommon aspect. Therefore, the development of the new fish products described in the present study was carried out considering the market study data, as well as the importance of using new fish species in familiar products. For example, the comber pastries were presented as a reformulation of a typical Portuguese product (cod pastries), adjusted for more traditional consumers. Additionally, the need to increase the snacks, salad ingredients, or side dishes offered (dehydrated piper gurnard) was also considered. Regardless of these needs, the formulation of dehydrated piper gurnard was intended to improve the traditional Portuguese processing of fish drying, in which salt is added and the drying process is performed by sun drying in open air, which is subject to all forms of contamination (e.g., pollution, flies). The need to increase the offer of ready-to-fry breaded products (boarfish) and fish-based pâtés (blue jack mackerel) was also considered. 

Pâté is a value-added food that can be developed with meat (e.g., liver from goose or pig) or fish [28]. However, the fish species used in pâté formulation are often those with significant commercial value, promoting their overexploitation, which needs to be avoided [28]. In addition to using low-value fish species, the pâté developed in the present study also offers an advantage for consumers’ health—the fish cooking process through smoking. Smoking is a type of preservation method that provides both antimicrobial smoke chemicals (e.g., formaldehydes, phenols) and reduced water activity, with the addition of giving an attractive color and flavor to the fish, improving the final product both nutritionally and sensorially [27].

The black seabream ceviche was developed to maintain the nutritional content in one of the developed fish products to adapt in new diets based on raw foods and new gastronomic trends. 

According to the initial survey referred to in this study, the processed fish products are present in consumers’ diets due to their practical and quick preparation. Therefore, three of the fish products developed in this study were ready-to-eat, and the boarfish and comber pastries only needed to be fried, reducing the consumer’s time spent on their preparation. 

### 3.3. New Fish-Based Food Product Acceptance—Hedonic Tests

#### 3.3.1. Black Seabream Ceviche

**Figure 2:**
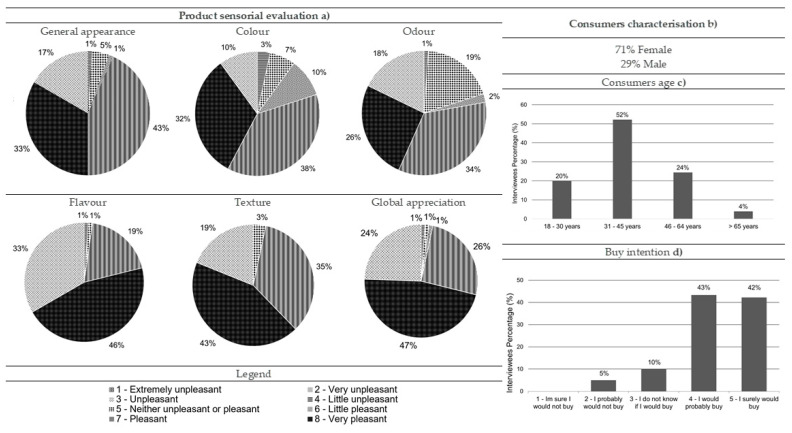
Results from the black seabream ceviche consumer acceptance survey: (**a**) sensorial evaluation (product’s general appearance, colour, odour, flavour, texture and global appreciation), (**b**) consumers characterisation (gender percentage), (**c**) consumers age and (**d**) buy intention (*n* = 90).

Most of consumers who performed the hedonic tests for black seabream ceviche were women (71%), and 52% were aged between 31 and 45 (Figure 2b,c, respectively). Regarding this product, 33% and 32% of the consumers considered its appearance and color very pleasant, respectively, while 34% rated its odor as pleasant (Figure 2a). Regarding flavor, texture, and global appreciation, consumers found these three descriptors very pleasant (46%, 43%, and 47%, respectively) (Figure 2a). The data also showed that most of the consumers would probably (43%) or certainly (42%) buy it (Figure 2d).

**Figure 3:**
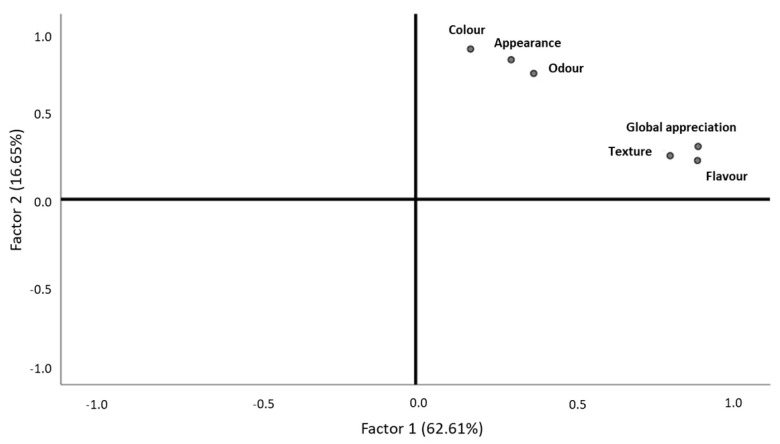
Behavior of black seabream ceviche descriptors, according to consumer classifications.

After the validation of the sample adequation (KMO = 0.798, Bartlett, *p*-value < 0.001), it was clear that black seabream ceviche texture, global appreciation, and flavor were related to each other (Figure 3). These descriptors belonged to the first factor, which explained 62.61% of the data variability and was labelled as “touch, taste, and overall sensory experience” (Figure 3, Table 4). The second factor, which explained 16.65% of the total data variability, was represented by color, appearance, and odor, which had less significance for the products’ description by consumers and was labelled as “sight and smell” (Figure 3, Table 4). These two groups characterized the product, although they did not have opposite behaviors (Figure 3). Consumers who rated texture, flavor and global appreciation with higher values considered these descriptors as the most important for black seabream ceviche characterization (Figure 3). Those who classified color with higher values also did so for appearance and odor (Figure 3). 

Both genders of all ages had the same pattern regarding the descriptors with greater importance, such as texture, flavor, and odor, with these descriptors being decisive for the products’ global appreciation by all consumers (Table 5). 

In fact, flavor was one of the consumers’ favorite attributes, as well as texture, with a “very pleasant” classification, followed by the appearance, color, and odor, classified as “pleasant” (Figure 2a). Black seabream ceviche was more accepted by women, who gave higher scores to flavor and purchase intention (middle rank = 48.83 and 48.82, respectively) than men (middle rank = 37.31 and 37.33, respectively; Mann–Whitney, *p*-value = 0.041 for both flavor and purchase intention). No statistical differences were reported when comparing age ranges (Kruskal-–Wallis, *p*-value > 0.05).

#### 3.3.2. Dehydrated Piper Gurnard

**Figure 4:**
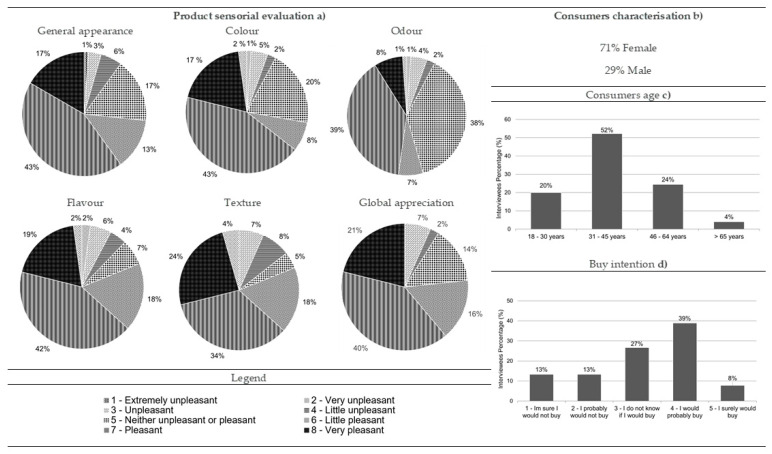
Results from the dehydrated piper gurnard consumer acceptance survey: (**a**) sensorial evaluation (product’s general appearance, colour, odour, flavour, texture and global appreciation), (**b**) consumers characterisation (gender percentage), (**c**) consumers age and (**d**) buy intention (*n* = 90).

Most of the consumers who performed the hedonic testes for dehydrated piper gurnard were women (71%), and 52% were aged between 31 and 45 (Figure 4b,c, respectively). The results revealed that all the descriptors were mainly considered “pleasant” by the consumers, with 43% for dehydrated piper gurnard appearance and color, 39% for odor, 42% for flavor, 34% for texture, and 40% for its global appreciation (Figure 4a). Concerning the purchase intention, 47% of consumers would probably or surely buy this product if it were on the market (Figure 4d). The levels of acceptance of this product could be increased if the conservation methods were improved, since they were not optimized during this formulation, which may have led to a loss of “crunchiness”or a gain in humidity that impaired the sensory evaluation of the product.

**Figure 5:**
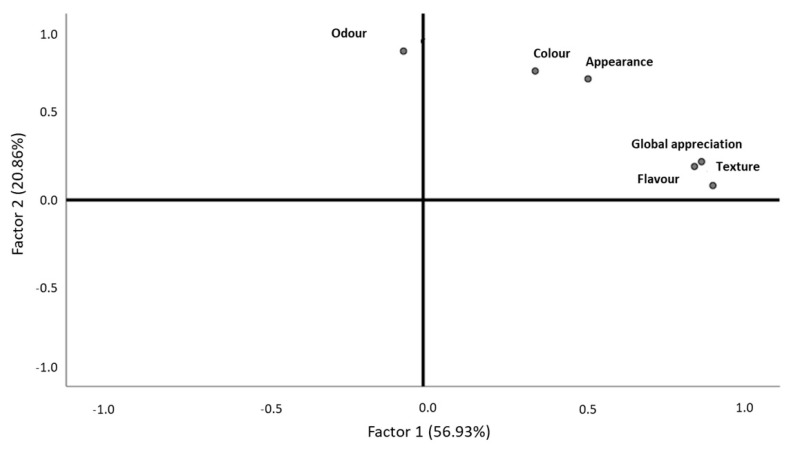
Behavior of dehydrated piper gurnard descriptors, according to consumer classifications.

After the validation of the sample adequation (KMO = 0.709, Bartlett, *p*-value < 0.001), the results revealed that dehydrated piper gurnard texture, global appreciation, and flavor were grouped together and belonged to the first factor, which explained most of the data variability (56.93%) and was labelled as “touch, taste, and overall sensory experience” (Figure 5, Table 6). The second factor, formed by odor, color, and appearance, explained 20.86% of the data variability and was labelled as “sight and smell” (Figure 5, Table 6). However, odor clearly differentiated itself from the other descriptors, assuming a differentiation role in this product (Figure 5). Therefore, these two groups were the ones that differentiated this product, although they did not have opposite behaviors (Figure 5). With this data, it is clear that those who classified texture with higher values also did so for overall appreciation and flavor, with these being the descriptors with most importance for consumers (Figure 5). Odor was the second most important descriptor, as it was the one that most contributed to the second axis characterization and so was responsible for another large part of the data variability (Figure 5). Those who classified color with higher values also did so for appearance (Figure 5). Not all descriptors related to each other with strong intensity, such as odor to flavor and global appreciation to odor, which correlated but with weaker intensity when compared to the other correlations (Table 5). The purchase intention was not statistically different among genders (Man–Whitney, *p*-value > 0.05) and age ranges (Kruskal–Wallis, *p*-value > 0.05).

#### 3.3.3. Fried Boarfish

**Figure 6:**
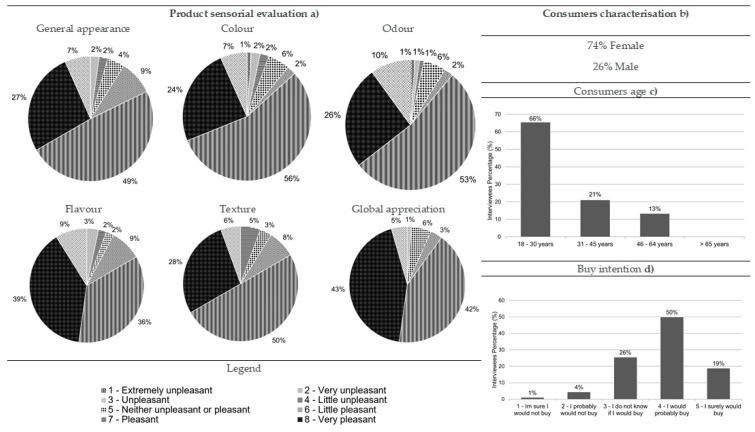
Results from the fried boarfish consumer acceptance survey: (**a**) sensorial evaluation (product’s general appearance, colour, odour, flavour, texture and global appreciation), (**b**) consumers characterisation (gender percentage), (**c**) consumers age and (**d**) buy intention (*n* = 90).

Most of the consumers who performed the hedonic testes for fried boarfish were women (74%), and 66% were aged between 18 and 30 (Figure 6). Fried boarfish is a product that was well accepted by consumers as all the descriptors were considered essentially very nice or pleasant (Figure 6). The fried boarfish’s general appearance was considered pleasant (49%), as were its color (56%), odor (53%), and texture (50%) (Figure 6). This product’s flavor and global appreciation were mostly considered very pleasant (39% and 43%, respectively) (Figure 6). In addition, the data also showed that 50% of the consumers would probably buy it (Figure 6).

**Figure 7:**
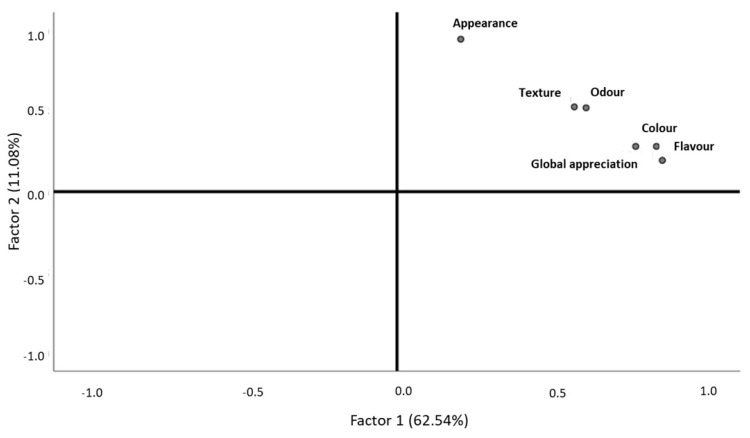
Behavior of fried boarfish descriptors, according to consumer classifications.

After the validation of the sample adequation (KMO = 0.859, Bartlett, *p*-value = 0.000), the results revealed that 62.54% of the data variability was explained by odor, texture, color, flavor, and global appreciation, which belonged to the first factor that was labelled as “all senses and overall sensory experience” (Figure 7, Table 7). The second factor was formed by appearance, which explained 11.08% of data variability and was labelled as “sight” (Figure 7, Table 7). Although the odor and texture were closer to the first factor, they were represented in the middle of the quadrant, revealing that they may not differentiate much either in terms of color, global appreciation, and flavor, or in terms of appearance (Figure 7). These two groups allowed for the differentiation of the fried boarfish, although they did not have opposite behaviors (Figure 7). Consumers who classified texture with higher values also did so for odor (Figure 7). Those who classified flavor with higher values also did so for color and global appreciation, these being the descriptors with most importance to consumers (Figure 7). The product’s appearance had almost no association with the color, global appreciation, or flavor of the fried boarfish. Therefore, the sensory evaluation of the product’s external aspect did not show any relevance to the evaluation of its color, global appreciation, or flavor (Figure 7). All the descriptors were statistically significant with intense correlation with one another (Table 5). No statistical differences were reported between descriptor classifications and purchase intention when comparing genders (Man–Whitney, *p*-value > 0.05). The purchase intention was statistically different between age ranges (Kruskal–Wallis, *p*-value = 0.037), where the older consumers liked the product more than the younger ones (Games–Howell, *p*-value = 0.005).

#### 3.3.4. Comber Pastries

**Figure 8:**
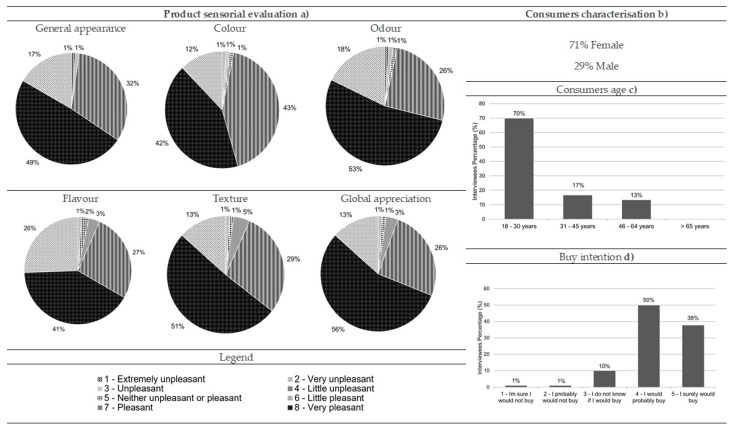
Results from the comber pastry consumer acceptance survey: (**a**) sensorial evaluation (product’s general appearance, colour, odour, flavour, texture and global appreciation), (**b**) consumers characterisation (gender percentage), (**c**) consumers age and (**d**) buy intention (*n* = 90).

Most of the consumers who performed the hedonic testes for comber pastries were women (71%), and 70% were aged between 18 and 30 (Figure 8b,c, respectively). In general, comber pastries were considered very pleasant by the consumers in a quantity of 49% for its general appearance, 42% for color, 53% for odor, 41% for flavor, 51% for texture, and 56% for global appreciation (Figure 8a). Regarding the purchase intention of this product, the results revealed that most of the consumers would probably or definitely buy it (50% and 38%, respectively) (Figure 8d).

**Figure 9:**
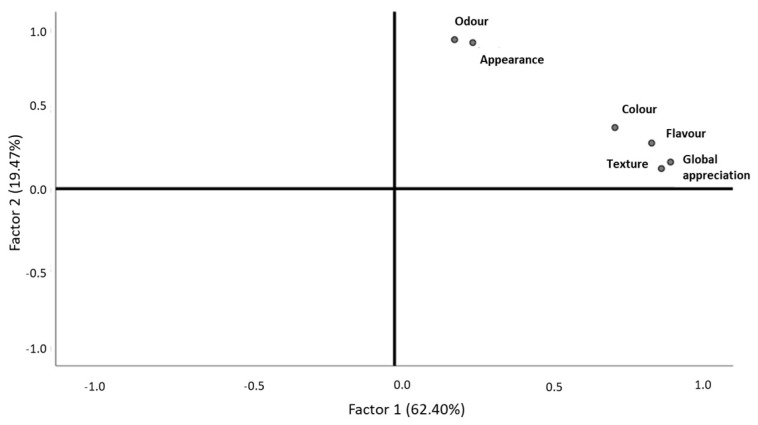
Behavior of comber pastry descriptors, according to consumer classifications.

After the validation of the sample adequation (KMO = 0.799, Bartlett, *p*-value = 0.000), it was noticed that comber pastries’ color, flavor, texture, and global appreciation were related to each other and helped to explain 62.40% of the data variability, belonging to the first factor that was labelled as “touch, taste, vision, and overall sensory experience” (Figure 9, Table 8). The second factor, which explained 19.47% of the data variability, is characterized by the odor and appearance and was labelled as “sight and smell” (Figure 9, Table 8). The consumers who ranked the appearance with higher values also did so for odor (Figure 9), whereas those who classified flavor with higher values also did so for texture, color, and global appreciation, with these being the descriptors with most importance for consumers (Figure 9). These two groups allowed for the differentiation of the comber pastries, although they did not reveal opposite behavior (Figure 9). The comber pastries’ odor and appearance had no association, especially with the global appreciation and texture, revealing that the visual aspect and the olfactory senses were not relevant in the product’s general evaluation and texture (Figure 9). 

All the descriptors had a statistically significant correlation with each other (Table 5). Only the correlation between texture and odor, as well as between global appreciation and odor, showed an association with a lower intensity (Table 5). The purchase intention was not statistically different among genders (Mann–Whitney, *p*-value > 0.05) and age ranges (Kruskal–Wallis, *p*-value > 0.05). 

#### 3.3.5. Smoked Blue Jack Mackerel Pâté

**Figure 10:**
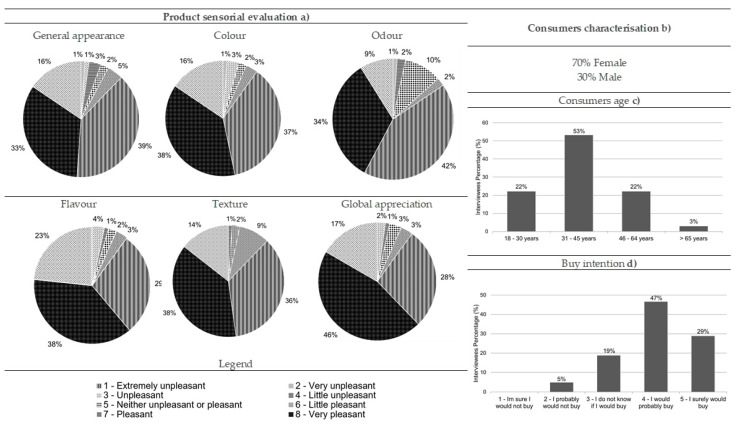
Results from the smoked blue jack mackerel pâté consumer acceptance survey: (**a**) sensorial evaluation (product’s general appearance, colour, odour, flavour, texture and global appreciation), (**b**) consumers characterisation (gender percentage), (**c**) consumers age and (**d**) buy intention (*n* = 90).

Most of the consumers who performed the hedonic testes for smoked blue jack mackerel pâté were women (70%), and 53% were aged between 31 and 45 (Figure 10b,c, respectively). Overall, this product was very well accepted by consumers. Regarding general appearance and odor, these descriptors were mostly classified as “pleasant” (39% and 42%, respectively) (Figure 10a). Color, flavor, texture, and global appreciation were considered by consumers as “very pleasant” attributes for this product (38%, 38%, 38%, and 46%, respectively) (Figure 10a). If the product was on sale in the market, most consumers indicated that they would probably or definitely buy it (47% and 29%, respectively) (Figure 10d).

**Figure 11:**
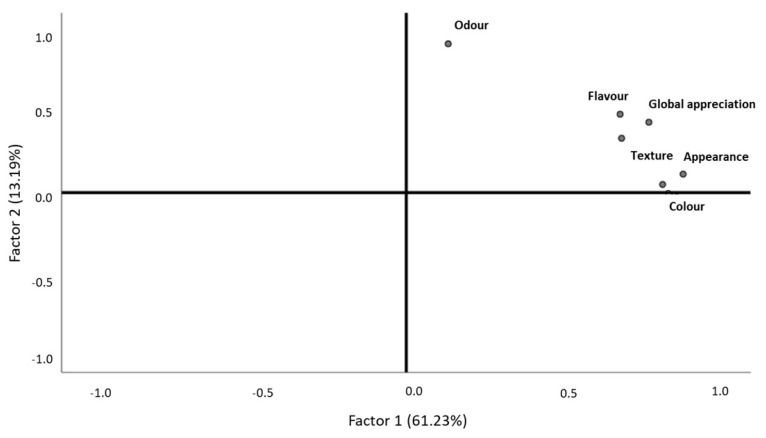
Behavior of smoked blue jack mackerel pâté descriptors, according to consumer classifications.

After the validation of the sample adequation (KMO = 0.795, Bartlett, *p*-value = 0.000), the results revealed that smoked blue jack mackerel pâté texture, global appreciation, appearance, color, and flavor were correlated with each other, characterizing the first factor that explained 61.23% of the data variability and was labelled as “touch, taste, sight, and overall sensory experience” (Figure 11, Table 9). The second factor, which explained 13.19% of the data variability, was characterized by odor and was labelled as “smell” (Figure 11, Table 9). Odor clearly differentiated itself from the other descriptors, assuming a differentiation role in smoked blue jack mackerel pâté (Figure 11). Therefore, these two groups were the ones that differentiated the product, although they did not have opposite behaviors (Figure 11). Consumers who classified texture with higher values also did so for global appreciation, appearance, color, and flavor, with these being the descriptors with most importance for consumers (Figure 11). Odor followed as the second most important descriptor, as it was the descriptor that most contributed for the second axis characterization and as such was responsible for another large part of the data variability (Figure 11). As mentioned for the remaining products with the exception of black seabream ceviche, odor revealed no association with other descriptors (Figure 11). In the case of the pâté, such a null association was found between its texture, appearance, and color, revealing that its visual aspect and texture did not show any relevance in the evaluation of the olfactory experience that the pâté offered to consumers (Figure 11). All the descriptors related to each other significantly and with strong intensity (Table 5). The purchase intention was not statistically different among genders (Mann–Whitney, *p*-value > 0.05) or age ranges (Kruskal–Wallis, *p*-value > 0.05). 

#### 3.3.6. Global Analysis

Considering the data as a whole, most of the developed food products showed statistically significant correlations between all the descriptors under study (Table 5). These results were expected considering that the perception and evaluation of food is an inherently multisensory experience, its pleasantness being influenced by food appearance, smell, taste, oral texture, and even by the sound that it makes in the mouth when it is eaten [29]. Therefore, the flavor perception is influenced by different factors including changes in viscosity, color cues, and interaction between oral texture and both olfactory and gustatory cues [29], whereas the color evaluation is an indicator of edibility, flavor identity, and intensity [30]. Therefore, the fish products developed in the present study had high acceptability by the consumers revealing their market potential. However, besides the “food-internal stimuli,” which is when flavor and taste are influenced by appearance, texture, and other descriptors, there are also “food-external stimuli” [31]. This stimulus regards health information, societal influence, and availability of certain foods that can affect liking of food products [31]. Thus, considering that consumers increasingly seek healthy food without food additives that are both quick and easy to prepare (as concluded by the initial market survey of this investigation), the products developed may offer new alternatives in addition to those existing in the market. Additionally, fishing is almost an immemorial activity on the Portuguese coast, always linked to the geographical position of Portugal and its contours, where fish has an added importance due to the gastronomic heritage of this country [32]. However, the demand on fish supply has been rising due to the rapid growth of the world’s population, favoring an imbalance with the demand of fishery products [33]. Consequently, the overexploitation or depletion of the world´s fish stocks increases, revealing the need to search for available fish species underutilized in human food that can be a major source of nutrients, including lipids and fatty acids [33]. Therefore, considering our data from the hedonic evaluation of the fish products developed in the present study, these species can be an alternative to commercialized fish species. In addition, the fish species valorization would increase the fisherman’s income, because fish that would be discarded on the high sea would have some commercial value. However, it must be noticed that the consumer dimension under study is not representative of the Portuguese population, and further studies on fish consumption and acceptance of reformulated products are necessary to allow the extrapolation of the data. Nonetheless, the present study provides some insight on this field and can be a valuable contribution for further studies on new fish product development and the characterization of fish consumption in the center of Portugal.

## 4. Conclusions

The present study allowed us to achieve great findings on interviewees’ habits of fish consumption, as well as their marine sustainability conscience, namely on underexploited and underutilized fish valorization and the main attributes for their consumption. According to the initial survey data, it was also possible to understand the receptivity to new fish products, preferably frozen, ready to eat, or to be prepared in the oven, which need to be properly promoted. The knowledge on consumers’ interests acquired with the data from the initial survey allowed for the development of new fish products, with the addition or substitution of the fish species under study through the reformulation of existing products that are familiar to the consumer. These products have a clean label, great acceptability, and purchase intention by the consumers. The results from hedonic tests revealed their potential application by companies to increase their revenue due to low or absent commercial value of discarded fish species. In fact, consumer evaluation revealed that they probably would buy all the developed products, with their global appreciation classified mainly as “very pleasant,” except for the dehydrated piper gurnard, which was classified as “pleasant.” In addition, the consumers favored the texture and flavor of the black seabream ceviche and dehydrated piper gurnard, with the addition of color for the comber pastries. The flavor and color of the fried boarfish were the most important features for consumers, with the addition of texture and appearance in the smoked blue jack mackerel pâté. Therefore, considering all our data and the need to avoid fish overexploitation, the species in this study revealed to be a great alternative to commercialized species commonly used in fish products. However, more research is needed to study Portuguese fish consumption, as well as their opinion about the inclusion of unexploited fish in their diet.

As future perspectives, it is intended to study and extend the shelf-life of the developed products through appropriate and sustainable packaging and maintaining the clean label.

## Figures and Tables

**Table 1:** Results of market survey concerning consumers’ gender, age, education level, and occupation (*n* = 155).

Baseline Characteristics	Survey Respondents (%)
Gender	
Male	31%
Female	69%
Age	
18–30	30%
31–45	42%
46–64	26%
>65	2%
Education Level	
Basic education	4%
High school	11%
Graduation	21%
Master’s degree	31%
PhD	33%
Occupation	
Student	17%
Employee	57%
Self-employed	6%
Retired	2%
Unemployed	2%
Research fellow	15%
Teacher	1%

**Table 2:** Results of the survey concerning the importance and frequency of fish consumption and its main reason, as well as the presence of fish with low or absent commercial value in the consumers diet (*n* = 155).

Questions	Survey Respondents (%)
1. Do you consider fish consumption to be important?	
Not very important	1%
Indifferent	1%
Important	26%
Very important	72%
1.1. If so, what are the main attributes you valorize in fish consumption?	
Fatty acids/omegas	79%
Protein	7%
Flavor	6%
Low-fat	6%
Low-sugar	1%
High metal content	1%
2. How often do you eat fish?	
<Once a month	1%
Two times a month	9%
Every week	44%
More than once a week	43%
Occasionally	3%
2.1 Do you know of or consume any kind of fish with low or no commercial value?	
Yes	48%
No	22%
Maybe	30%

**Table 3:** Results of the survey concerning consumers’ consumption habits of processed fish products (*n* = 155), the reason for their consumption (*n* = 117), their receptivity to these products’ innovation (*n* = 155), what type of innovative products the consumer would like (*n* = 124), the significance of fish species valorization (*n* = 155), and the importance of new product formulation with their inclusion and promotion (*n* = 155).

Questions	Survey Respondents (%)
3. Do you consume processed fish?	
Yes	75%
No	25%
3.1 If so, why do you consume these processed foods?	
Practical and quick meals	38%
Tasty products	20%
Low-price	18%
Nutritionally rich	3%
Does not need to be cooked	10%
Long shelf-life	11%
4. Are you receptive to new processed fish products?	
Yes	86%
No	6%
Maybe	8%
4.1 If you answered yes in the previous answer, please indicate which ones:	
Oven products	40%
Ready-to-eat products	6%
Frozen products	3%
All previous answers	51%
5. Do you think it is important to valorize fish species without commercial value?	
Not important	1%
Little importance	1%
Indifferent	1%
Important	36%
Very important	61%
6. Do you consider it important that fish valorization be done through the formulation of new food products?	
Not important	1%
Little importance	1%
Indifferent	9%
Important	54%
Very important	35%
7. What is the importance of promoting this type of product that aims at the valorization of species without commercial value?	
Not important	1%
Little importance	0%
Indifferent	1%
Important	41%
Very important	57%

**Table 4:** Rotated component matrix for each black seabream ceviche descriptor.

Descriptor	Component	
	1	2
	Touch, Taste, and Overall Sensory Experience	Sight and Smell
Appearance	0.299	0.837
Color	0.172	0.901
Odor	0.370	0.755
Flavor	0.883	0.232
Texture	0.797	0.261
Global Appreciation	0.885	0.316

Extraction method; principal component analysis; rotation method; varimax with Kaiser normalization; rotation converged in three iterations.

**Table 5:** Correlation with Spearman’s non-parametric test between all descriptors (appearance, color, odor, flavor, texture, and global appreciation) for each fish product developed (*n* = 90).

Black Seabream Ceviche							
Population Group	Descriptor	*p*-Value					
		Appearance	Color	Odor	Flavor	Texture	Global Appreciation
All	Appearance	-	0.000 *	0.000 *	0.000 *	0.000 *	0.000 *
	Color	0.000 *	-	0.000 *	0.000 *	0.000 *	0.000 *
	Odor	0.000 *	0.000 *	-	0.000 *	0.000 *	0.000 *
	Flavor	0.000 *	0.000 *	0.000 *	-	0.000 *	0.000 *
	Texture	0.000 *	0.000 *	0.000 *	0.000 *	-	0.000 *
	Global Appreciation	0.000 *	0.000 *	0.000 *	0.000 *	0.000 *	-
Dehydrated Piper Gurnard							
All	Appearance	-	0.000 *	0.000 *	0.000 *	0.000 *	0.000 *
	Color	0.000 *	-	0.000 *	0.002 *	0.000 *	0.000 *
	Odor	0.000 *	0.000 *	-	0.026 *	0.566	0.013 *
	Flavor	0.000 *	0.000 *	0.026 *	-	0.000 *	0.000 *
	Texture	0.000 *	0.000 *	0.566	0.000 *	-	0.000 *
	Global Appreciation	0.000 *	0.000 *	0.013 *	0.000 *	0.000 *	-
Fried Boarfish							
All	Appearance	-	0.000 *	0.000 *	0.000 *	0.000 *	0.000 *
	Color	0.000 *	-	0.000 *	0.000 *	0.000 *	0.000 *
	Odor	0.000 *	0.000 *	-	0.000 *	0.000 *	0.000 *
	Flavor	0.000 *	0.000 *	0.000 *	-	0.000 *	0.000 *
	Texture	0.000 *	0.000 *	0.000 *	0.000 *	-	0.000 *
	Global Appreciation	0.000 *	0.000 *	0.000 *	0.000 *	0.000 *	-
Comber Pastries							
All	Appearance	-	0.000 *	0.000 *	0.004 *	0.009 *	0.001 *
	Color	0.000 *	-	0.000 *	0.000 *	0.000 *	0.000 *
	Odor	0.000 *	0.000 *	-	0.010 *	0.044 *	0.041 *
	Flavor	0.004 *	0.000 *	0.010 *	-	0.000 *	0.000 *
	Texture	0.009 *	0.000 *	0.044 *	0.000 *	-	0.000 *
	Global Appreciation	0.001 *	0.000 *	0.041 *	0.000 *	0.000 *	-
Smoked Blue Jack Mackerel Pâté							
All	Appearance	-	0.000 *	0.000 *	0.000 *	0.000 *	0.000 *
	Color	0.000 *	-	0.000 *	0.000 *	0.000 *	0.000 *
	Odor	0.000 *	0.000 *	-	0.000 *	0.000 *	0.000 *
	Flavor	0.000 *	0.000 *	0.000 *	-	0.000 *	0.000 *
	Texture	0.000 *	0.000 *	0.000 *	0.000 *	-	0.000 *
	Global Appreciation	0.000 *	0.000 *	0.000 *	0.000 *	0.000 *	-

* Statistically significant correlation between descriptors; - not applicable.

**Table 6:** Rotated component matrix for each dehydrated piper gurnard descriptor.

Descriptor	Component	
	1	2
	Touch, Taste, and Overall Sensory Experience	Sight and Smell
Appearance	0.513	0.722
Color	0.349	0.769
Odor	−0.061	0.887
Flavor	0.845	0.200
Texture	0.902	0.086
Global Appreciation	0.867	0.228

Extraction method; principal component analysis; rotation method; varimax with Kaiser normalization; rotation converged in three iterations.

**Table 7:** Rotated component matrix for each fried boarfish descriptor.

Descriptor	Component	
	1	2
	All Senses and Overall Sensory Experience	Sight
Appearance	0.206	0.944
Colour	0.773	0.280
Odour	0.612	0.519
Flavour	0.859	0.194
Texture	0.574	0.525
Global appreciation	0.840	0.280

Extraction method; Principal component analysis; Rotation method; Varimax with Kaiser normalization; Rotation converged in 3 iterations.

**Table 8:** Rotated component matrix for each comber pastry descriptor.

Descriptor	Component	
	1	2
	Touch, Taste, Vision, and Overall Sensory Experience	Sight and Smell
Appearance	0.257	0.917
Color	0.723	0.384
Odor	0.197	0.935
Flavor	0.843	0.286
Texture	0.875	0.126
Global Appreciation	0.905	0.167

Extraction method; principal component analysis; rotation method; varimax with Kaiser normalization; rotation converged in three iterations.

**Table 9:** Rotated component matrix for each smoked blue jack mackerel pâté descriptor.

Descriptor	Component	
	1	2
	Touch, Taste, Sight, and Overall Sensory Experience	Smell
Appearance	0.893	0.114
Color	0.826	0.050
Odor	0.135	0.920
Flavor	0.689	0.485
Texture	0.694	0.336
Global Appreciation	0.782	0.435

Extraction method; principal component analysis; rotation method; varimax with Kaiser normalization; rotation converged in three iterations.
